# Peste des petits ruminants outbreaks in White Nile State, Sudan

**DOI:** 10.4102/ojvr.v82i1.897

**Published:** 2015-08-21

**Authors:** Osama M. Ishag, Intisar K. Saeed, Yahia H. Ali

**Affiliations:** 1 Rabak Veterinary Research Laboratory, White Nile state, Sudan; 2 Department of Virology, Veterinary Research Institute, Soba, Sudan

## Abstract

Eight outbreaks of peste des petits ruminants in sheep and goats were reported in White Nile State, Sudan, between 2008 and 2009. A mortality rate of 4.2% was reported across the different outbreaks. Clinically the disease was characterised by high fever, ocular and nasal discharge, pneumonia, ulceration of the mucous membranes, diarrhoea and death. The post-mortem findings included necrotic lesions in the mouth and gastrointestinal tract, and swollen, oedematous lymph nodes associated with the lungs and intestine. Of the 209 serum samples tested by competitive enzyme-linked immunosorbent assay, 113 (54%) were found positive. Peste des petits ruminants virus was confirmed in tissues, nasal swabs and blood samples by immunocapture enzyme-linked immunosorbent assay, reverse-transcription polymerase chain reaction and isolation of the virus in culture of lamb testicle cells.

## Introduction

Peste des petits ruminants (PPR) is an acute, highly contagious viral disease of sheep and goats (Diallo *et al.* 2007; Kwiatek *et al.* 2007). The PPR virus (PPRV) is classified as a member of the genus *Morbillivirus* in the family *Paramyxoviridae*. The genome, which consists of single-stranded negative-sense RNA of approximately 16 kb, encodes eight proteins, namely a nucleocapsid protein (N), a phosphoprotein (P), a matrix protein (M), a fusion protein (F), a haemagglutinin protein (H), a large polymerase protein (L) and two non-structural proteins (C and V) (Bailey *et al.* 2005; Singh *et al.* 2004). Genetic classification of PPRV has identified four lineages by partial sequencing of the F and N genes (Rossiter 2004).

The economic impacts of the disease are due to high morbidity and mortality. Morbidity of between 50% and 100% has been reported, and mortality of 20% – 100% (Roeder & Obi 1999; Singh & Prasad 2008). Lower mortality is common in endemic areas (Roeder & Obi 1999), whereas higher mortality is generally observed when PPRV infection is associated with other diseases, such as sheep and goat pox (Dhar *et al.* 2002). The mortality rate may vary between species and it has been reported that the case-fatality rate can reach 55% – 85% in goats, 10% in sheep and 50% in camels (Khalafalla *et al.* 2010; Radostits *et al.* 2007). High abortion rates have been reported in goats (Abubakar, Ali & Khan 2008). PPR is considered to be the main constraint to increasing sheep and goat production in countries where it is endemic and the most economically important viral disease of small ruminants where it occurs (Nanda *et al.* 1996; Rossiter 2004).

Diagnosis of PPR is based on clinical examination, post-mortem lesions and laboratory tests. The main symptoms of the disease are fever, pneumonia with nasal and ocular discharges, and diarrhoea. In addition to viral isolation, laboratory tests to detect the antigen, genome and antibodies are available. The competitive enzyme-linked immunosorbent assay (cELISA) test used for detection of PPR antibodies is very sensitive and specific (Libeau *et al.* 1995). Libeau *et al.* (1994) also developed an immunocapture ELISA (IcELISA) for detection of PPRV antigen, which is reliable and can be used to differentiate PPR from rinderpest.

PPR historically occurred in West Africa, the Middle East and South Asia, and more recently has also been reported from northern sub-Saharan Africa. The distribution of PPR has expanded during the recent past, with the disease detected for the first time in Uganda and Kenya in 2007, in Tanzania in 2009, in Morocco in 2008 (Kwiatek *et al.* 2011), in Tunisia in 2007 (Ayari-Fakhfakh *et al.* 2011) and in Angola in 2012 (World Organisation for Animal Health [OIE] 2012).

In Sudan, PPR was first reported by El Hag (1973) and since then regular outbreaks have been reported, mainly in sheep and goats (Kwiatek *et al.* 2011). However, it has not previously been confirmed in White Nile State. This report describes outbreaks that occurred amongst sheep and goats there in 2008/2009.

## Outbreak investigation

**FIGURE 1:**
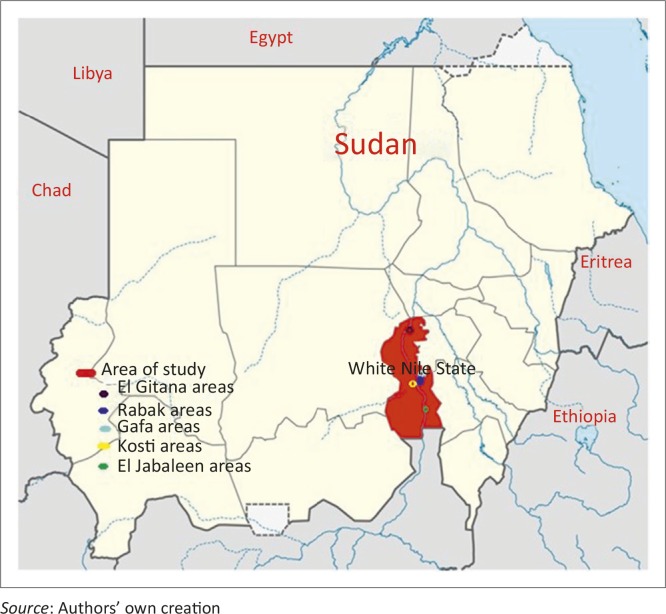
Map of the Sudan, with the area of study indicated.

White Nile State is situated in the south of Northern Sudan, (13 16’27”N; 33 26’59”E) and has a total area of 39 701 km^2^ (Figure 1). The population of the state is estimated at 1.73 million inhabitants, about two-thirds of whom live in rural areas (Central Bureau of Statistics 2009). The small ruminant population is estimated at about 1.8 million sheep and close to 1.7 million goats, with a rural household having, on average, eight goats and one sheep. The main production systems are nomadic pastoralism and agro-pastoralism. Small ruminants often feed in open pastures on natural grazing, where they are herded by children or women. Livestock and its production is considered an important source of protein, livestock provide saving and insurance for rural households and 32% of farm income to rural households in White Nile state.

Over a period of 2 years (2008–2009), outbreaks of a fatal disease in sheep and goats were reported to veterinary authorities in White Nile State. The first case of the disease was reported to the Rabak veterinary clinic. The animals presented with signs of pyrexia with inappetence, discharge from the eyes, nose and mouth, diarrhoea and death. Symptomatic treatment was given to sick animals without significant response. The disease then also occurred in different parts of the state. In all areas where the disease was reported that were visited, animals were examined, clinical signs and mortality rates were recorded, and epidemiological data were collected. Samples collected included 209 serum samples (38 from goats and 171 from sheep), 20 whole-blood samples and 80 swabs taken from sheep and goats with and without signs of PPR. Twenty tissue samples from lung and lymph nodes were also collected, homogenised as a 20% suspension in phosphate buffered saline and stored at -20 °C until use.

Antibody detection using a cELISA kit (CIRAD, Montpellier) and antigen detection using an IcELISA kit (CIRAD, Montpellier) were performed according to the manual provided with the kits. Virus isolates were prepared from lamb testicle cells in a disposable tissue culture flask. For molecular diagnosis, RNA was extracted from tissue samples using an RNA extraction kit (RNeasy Mini kit, Qiagen, Germany) according to the manufacturer’s recommended protocol. The OneStep RT-PCR kit (Qiagen, Germany) was used for amplification of the N protein, following the manufacturer’s recommended protocol. Reverse-transcription polymerase chain reaction (RT-PCR) amplified the N protein gene using primers as described by Couacy-Hymann *et al.* (2002).

Clinical signs observed varied between individual animals, but the most commonly observed signs were sudden death of apparently healthy animals, especially in the younger age groups, serous discharge from the eyes, nose and mouth, which later became thick and yellowish, epithelial necrosis of the lips, inner cheeks and upper surface of the tongue, watery foul-smelling diarrhoea, disturbed breathing, dyspnoea and sneezing in an attempt to clear the nose, and death (Table 1). The overall mortality rate due to PPR was 4.2%, as shown in Table 2.

**TABLE 1:** Clinical signs and date of reported cases in White Nile State, Sudan.

Area	Date	Clinical signs
Aboshateen	24 April 2008	Nasal and ocular discharge, diarrhoea, erosion in gum
EL Jazera mosran	05 May 2008	Pneumonia
Rabak Villages 1	12 June 2008	Pneumonia
El diwam villages	12 July 2008	Nasal and ocular discharge
EL Jazera Aba	10 August 2008	Pneumonia
EL kawoa	24 October 2008	Pneumonia
Joda	04 November 2008	Pneumonia
Kosti villages	09 November 2008	Mucopurulent nasal discharge, diarrhoea
Gafa	12 November 2008	Diarrhoea, death
Gala EL beat	16 November 2008	Death in lambs, ocular and nasal discharge, diarrhoea
Taeba	17 November 2008	Pneumonia, foul-smelling diarrhoea
EL zeelat (elsiferaia)	06 January 2009	Nasal discharge, diarrhoea, abortion, sudden death in lambs and adults
Gafa (eljeebal elboeut)	13 January 2009	Mucopurulent nasal discharge, foul-smelling diarrhoea, death
Rabak villages 2	03 February 2009	Nasal discharge
Rabak villages 3	04 February 2009	Pneumonia

**TABLE 2:** Number of animals affected and mortality rate during the 2008–2009 outbreak of peste des petits ruminants in White Nile State, Sudan.

Area	Total number of animals	Mortality	
		*n*	%
Aboshateen	250	50	20.0
Gafa	750	39	5.2
El Jabaleen areas (Joda, El Jazera mosran, Gala El beat)	350	100	28.6
El Jazera Aba	200	10	5.0
El zeelat (elsiferaia)	2000	50	2.5
Rabak villages (1, 2, 3)	750	10	1.3
Kosti villages	3000	50	1.7
Total	7300	309	4.2

At post-mortem examination, the main findings were necrotic and erosive mucosa inside the lips and on the dorsal surface of the tongue, and congestion of the lung and small intestine; the associated lymph nodes were congested, enlarged and soft.

As shown in Table 3, 113 serum samples were found positive for PPRV by cELISA, with more adult animals affected than young animals.

**TABLE 3:** Analysis of serum samples as tested by competitive enzyme-linked immunosorbent assay.

Result	Sheep		Goat		Total
	> 2 years	< 2 years	> 2 years	< 2 years	
Positive	55	38	15	5	113
Negative	58	20	16	2	96
Total	113	58	31	7	209

Using IcELISA, PPRV antigen was detected in 11% of swab samples, 35% of tissue samples and 30% of whole-blood samples (Table 4).

**TABLE 4:** Analysis of swab, tissue and whole-blood samples as tested by immunocapture enzyme-linked immunosorbent assay.

Sample	Number of sample tested	Positive		Negative	
		*n*	%	*n*	%
Tissue	20	7	35	13	65
Swab	80	9	11	71	89
Whole blood	20	6	30	14	70
**Total**	**120**	**22**	**18**	**98**	**82**

PPRV was isolated successfully in lamb testicle cells. Typical PPRV cytopathic effects (CPE) appeared on day 14 post inoculation and the final harvest of the infected cells was on day 25 post inoculation. Two further passages were made on lamb testicle cells.

Of the seven tissue samples (lungs and lymph nodes) tested by RT-PCR, all were found positive.

## Discussion

During 2008 and 2009 eight outbreaks of an unknown fatal disease, with symptoms of pneumonia and diarrhoea, were reported in sheep and goats in White Nile State, Sudan. Based on the epidemiological, clinicopathological and virological findings, the disease was confirmed to be PPR. Data collected from the field indicated that farmers had no knowledge about PPR before the outbreaks, although the disease had been reported in all states bordering White Nile State. Many of the rural population cross the state with their animals in search of green pastures and water and therefore contagious diseases such as PPR can be transmitted from sick to healthy animals when they mix whilst grazing and drinking.

Field and laboratory investigations indicated that the disease manifested as acute to peracute in the younger age groups. The features of the disease were discharge from the eyes, nose and mouth, necrotic stomatitis, diarrhoea and pneumonia, in line with symptoms that have been reported previously (Aiello & Mays 1998; Khan *et al.* 2008; Roeder & Obi 1999).

Abubakar *et al.* (2009) stated that the seroprevalence of PPR is high in sheep and goats older than 2 years, but Al-Majali *et al.* (2008), Ozkul *et al.* (2002) and Singh *et al.* (2004) reported that sheep and goats between 4 months and 2 years are more likely to be seropositive for PPR. The study showed that there was no significant difference in the number of seropositive sheep and goats older than 2 years than younger ones and more sheep were seropositive than goats; however, Megersa *et al.* (2011), reported a higher seroprevalence in goats than in sheep and linked it to higher fecundity in goats than in sheep. It was noted in this study that climatic factors had a marked influence on the spread of PPR, with most outbreaks occurring in the colder months, similar to reports by Durojaiye, Obi and Ojo (1983).

PPRV antigen was confirmed by IcELISA in samples that were taken from sick animals and at post-mortem examinations of animals that had recently died of PPR.

All tissue samples tested for the PPRV genome were found positive by means of RT-PCR. This result agrees with previous reports that described the high sensitivity of RT-PCR using NP3/NP4 primers for detection of PPR nucleic acids (Couacy-Hymann *et al.* 2009; Kumar *et al.* 2007). Isolation of PPRV in lamb testicle cell culture produced a CPE consisting of rounding of cells and syncytia formation at 14 days post inoculation. This finding is similar to that described by Hamdy *et al.* (1976) and Intisar (2002), although Intisar (2002) reported that growth of PPRV in lamb testicle cells was slower, with a CPE observed only 17–20 days post innoculation.

After PPRV had been diagnosed and confirmed to be the cause of this outbreak, a mass vaccination campaign was undertaken by the veterinary authority in White Nile State and quarantine and movement control were instituted to prevent spread of the disease.

The possibility that the severity of the disease observed in the outbreaks was due to the appearance of a new lineage (lineage IV) in Sudan was considered. However, an investigation of samples from various regions in Sudan, including a sample from these outbreaks, indicated a gradual replacement of lineage III by lineage IV from about mid 2000 (Kwiatek *et al.* 2011). The low mortality observed (see Table 2) suggests possible previous exposure, which may have rendered a relatively high proportion of the older animals immune, whereas young animals developed severe symptoms.

## References

[CIT0001] Abubakar M., Ali Q. & Khan H.A., 2008 ‘Prevalence and mortality rate of peste des petits ruminants (PPR): Possible association with abortion in goat’, *Tropical Animal Health and Production* 40, 317–321. http://dx.doi.org/10.1007/s11250-007-9105-2 18509938 10.1007/s11250-007-9105-2

[CIT0002] Abubakar M., Jamal S.M., Arshed M.J., Hussain M. & Ali Q., 2009, ‘Peste des petits ruminants virus (PPRV) infection: Its association with species, seasonal variations and geography’, *Tropical Animal Health and Production* 41(7), 1197–1202. http://dx.doi.org/10.1007/s11250-008-9300-9 19130284 10.1007/s11250-008-9300-9

[CIT0003] Aiello S.E. & Mays A., 1998, ‘Peste des petits ruminants’, in Aiello S.E. & Mays A. (eds.), *Merck veterinary manual*, 8th edn, pp. 539–541, White house Station, NJ.

[CIT0004] Al-Majali A.M., Hussain N.O., Amarin N.M. & Majok A.A., 2008, ‘Seroprevalence of, and risk factors for, peste des petits ruminants in sheep and goat in north Jordan’, *Preventive Veterinary Medicine* 85, 1–8. http://dx.doi.org/10.1016/j.prevetmed.2008.01.002 18291541 10.1016/j.prevetmed.2008.01.002

[CIT0005] Ayari-Fakhfakh E., Ghram A., Bouattour A., Larbi I., Gribaa-Dridi L., Kwiatek O. et al., 2011, ‘First serological investigation of peste-des-petits-ruminants and Rift Valley fever in Tunisia’, *Veterinary Journal* 187(3), 402–404. http://dx.doi.org/10.1016/j.tvjl.2010.01.007 10.1016/j.tvjl.2010.01.007 20167519

[CIT0006] Bailey D., Banyard A., Dash P., Ozkul A. & Barret T., 2005, ‘Full genome sequence of peste des petits ruminants virus, a member of the Morbillivirus genus’, *Virus Research* 110, 119–124. http://dx.doi.org/10.1016/j.virusres.2005.01.013 15845262 10.1016/j.virusres.2005.01.013

[CIT0007] Central Bureau of Statistics, 2009, *Sudan national baseline household survey 2009 North Sudan* – *tabulation report*, Central Bureau of Statistics, Khartoum.

[CIT0008] Couacy-Hymann E., Bodjo S.C., Koffi M.Y., Kouakou C. & Danho T., 2009, ‘The early detection of peste-des-petits-ruminants (PPR) virus antigens and nucleic acid from experimentally infected goats using RT-PCR and immunocapture ELISA techniques’, *Research in Veterinary Science* 87(2), 332–335. http://dx.doi.org/10.1016/j.rvsc.2009.03.002 19339027 10.1016/j.rvsc.2009.03.002

[CIT0009] Couacy-Hymann E. Roger F., Hurard C., Guillou J.P., Libeau G. & Diallo A., 2002, ‘Rapid and sensitive detection of peste des petits ruminants virus by a polymerase chain reaction assay’, *Journal of Virological Methods* 100, 17–25. http://dx.doi.org/10.1016/S0166-0934(01)00386-X 11742649 10.1016/s0166-0934(01)00386-x

[CIT0010] Dhar P., Sreenivasa B.P., Barrett T., Corteyn M., Singh R.P. & Bandyopadhyay S.K., 2002, ‘Recent epidemiology of peste des petits ruminants virus’, *Veterinary Microbiology Journal* 88, 153–159. http://dx.doi.org/10.1016/S0378-1135(02)00102-5 10.1016/s0378-1135(02)00102-5 12135634

[CIT0011] Diallo A., Minet C., Le Goff C., Berhe G., Albina A., Libeau G. et al., 2007, ‘The threat of peste des petits ruminants: Progress in vaccine development for disease control’, *Vaccine* 25, 5591–5597. http://dx.doi.org/10.1016/j.vaccine.2007.02.013 17399862 10.1016/j.vaccine.2007.02.013

[CIT0012] Durojaiye O.A., Obi T.U. & Ojo O., 1983, ‘Virological and serological diagnosis of peste des petits ruminants’, *Tropical Veterinarian* 1, 13–17.

[CIT0013] El Hag B.A., 1973, ‘A natural outbreak of rinderpest involving sheep, goat and cattle in Sudan’, *Bulletin of Epizootic Diseases of Africa* 12, 421–428. 4807688

[CIT0014] Hamdy F.M., Dardiri A.H., Nduaka O., Breese S.S. & Ihemelandu E.C., 1976, ‘Etiology of the stomatitis pneumoenteritis complex in Nigerian dwarf goats’, *Canadian Journal of Comparative Medicine* 40, 276–284. 826310 PMC1277766

[CIT0015] Intisar K.S., 2002, ‘Studies on peste des petits ruminants disease in Sudan’, MSc thesis, Dept of Virology, University of Khartoum.

[CIT0016] Khalafalla A.I., Saeed I.K., Ali Y.H., Abdurrahman M.B., Kwiatek O., Libeau G. et al., 2010, ‘An outbreak of peste des petits ruminants (PPR) in camels in the Sudan’, *Acta Tropica* 116, 161–165. http://dx.doi.org/10.1016/j.actatropica.2010.08.002 20707980 10.1016/j.actatropica.2010.08.002

[CIT0017] Khan H.A., Siddique M., Abubakar M., Arshad M.J. & Hussain M., 2008, ‘Prevalence and distribution of peste des petits ruminants virus infection in Pakistan’, *Small Ruminant Research* 79, 152–157. http://dx.doi.org/10.1016/j.smallrumres.2008.07.021

[CIT0018] Kumar C.S., Dhinakar Raj G., Thangavelu A. & Shaila M. S., 2007, ‘Performance of RT-PCR-ELISA for the detection of peste des petits ruminants virus’, *Small Ruminant Research* 72, 200–208. http://dx.doi.org/10.1016/j.smallrumres.2006.09.004

[CIT0019] Kwiatek O., Ali Y., Saeed I., Khalafalla A, Mohamed O., Abu Obeida A. et al., 2011, ‘Asian lineage of peste des petits ruminants virus, Africa’, *Emerging Infectious Diseases* 17, 1223–1231. http://dx.doi.org/10.3201/eid1707.101216 21762576 10.3201/eid1707.101216 PMC3381390

[CIT0020] Kwiatek O., Minet C., Grillet C., Hurard C., Carlsson E., Karimov B. et al., 2007, ‘Peste des petits ruminants (PPR) outbreak in Tajikistan’, *Journal of Comparative Pathology* 136(2–3), 111–119. http://dx.doi.org/10.1016/j.jcpa.2006.12.002 17321539 10.1016/j.jcpa.2006.12.002

[CIT0021] Libeau G., Diallo A., Colas F. & Guerre L., 1994, ‘Rapid differential diagnosis of rinderpest and peste des petits ruminants using an immunocapture ELISA’, *Veterinary Record* 134, 300–304. http://dx.doi.org/10.1136/vr.134.12.300 8009788 10.1136/vr.134.12.300

[CIT0022] Libeau G., Perhaud C., Lancelot R., Colas F., Guerre L., Bishop D.H.L. et al., 1995, ‘Development of competitive ELISA for detecting antibodies to the peste des petits ruminants virus using a recombinant nucleoprotein’, *Research in Veterinary Science* 58, 50–55. http://dx.doi.org/10.1016/0034-5288(95)90088-8 7709061 10.1016/0034-5288(95)90088-8

[CIT0023] Megersa B., Biffa D., Belina T., Debela E., Regassa A., Abunna F. et al., 2011, ‘Serological investigation of peste des petits ruminants (PPR) in small ruminants managed under pastoral and agro-pastoral systems in Ethiopia’, *Small Ruminant Research* 97, 134–138. http://dx.doi.org/10.1016/j.smallrumres.2011.03.003

[CIT0024] Nanda Y.P., Chatterjee A., Purohit A.K., Diallo A., Innui K., Sharma R.N. et al., 1996, ‘The isolation of peste des petits ruminants virus from Northern India’, *Veterinary Microbiology* 51, 207–216. http://dx.doi.org/10.1016/0378-1135(96)00025-9 8870184 10.1016/0378-1135(96)00025-9

[CIT0025] Ozkul A., Akca Y., Alkan F., Barrett T., Karaoglu T., Dagalp S.B. et al., 2002, ‘Prevalence, distribution, and host range of peste des petits ruminants virus, Turkey’, *Emerging Infectious Diseases* 8(7), 708–712. http://dx.doi.org/10.3201/eid0807.010471 12095439 10.3201/eid0807.010471 PMC2730320

[CIT0026] Radostits O.M., Gay C.C., Hinchcliff K.W. & Constable P.D., 2007, *Veterinary medicine: A textbook of the diseases of cattle, horses, sheep, pigs and goats*, 10th edn., Saunders Elsevier, Philadephia, PA.

[CIT0027] Roeder P.L. & Obi T.U., 1999, *Recognizing peste des petites ruminants*, Food & Agriculture Organization of the United Nations, Rome (FAO Animal Health Manual series, no. 5).

[CIT0028] Rossiter P.B., 2004, ‘Peste des petits ruminants’, in Coetzer J.A.W. & Tustin R.C. (eds.), *Infectious diseases of livestock*, 2nd edn., vol. 2, pp. 660–672, Oxford University Press, Cape Town.

[CIT0029] Singh R.P., Bandyopadhyay S.K., Sreenivasa B.P. & Dhar P., 2004, ‘Production and characterization of monoclonal antibodies to peste des petits ruminants’, *Veterinary Research Communications* 28, 623–639. http://dx.doi.org/10.1023/B:VERC.0000042875.30624.67 15563110 10.1023/b:verc.0000042875.30624.67

[CIT0030] Singh B. & Prasad S., 2008, ‘Modelling of economic losses due to some important disease in goats in India’, *Agriculture Economics Research Review* 21, 297–302.

[CIT0031] World Organisation for Animal Health (OIE), 2012, *Peste des petits ruminants, Angola*, viewed on 15 October 2014, from http://www.oie.int/wahis_2/public/wahid.php/Reviewreport/Review?page_refer=MapFullEventReport&reportid=12408

